# Changes in lipid metabolism and capillary density of the skeletal muscle following low-intensity exercise training in a rat model of obesity with hyperinsulinemia

**DOI:** 10.1371/journal.pone.0196895

**Published:** 2018-05-02

**Authors:** Naoto Fujita, Saki Aono, Kohei Karasaki, Fumi Sera, Tomoyuki Kurose, Hidemi Fujino, Susumu Urakawa

**Affiliations:** 1 Department of Musculoskeletal Functional Research and Regeneration, Graduate School of Biomedical and Health Sciences, Hiroshima University, Minami-ku, Hiroshima, Japan; 2 Life and Medical Science Area, Health Sciences Discipline, Kobe University, Suma-ku, Kobe, Japan; Max Delbruck Centrum fur Molekulare Medizin Berlin Buch, GERMANY

## Abstract

Although exercise is effective in improving obesity and hyperinsulinemia, the exact influence of exercise on the capillary density of skeletal muscles remains unknown. The aim of this study was to investigate the effects of low-intensity exercise training on metabolism in obesity with hyperinsulinemia, focusing specifically on the capillary density within the skeletal muscle. Otsuka Long-Evans Tokushima fatty (OLETF) rats were used as animal models of obesity with hyperinsulinemia, whereas Long-Evans Tokushima Otsuka (LETO) rats served as controls (no obesity, no hyperinsulinemia). The animals were randomly assigned to either non-exercise or exercise groups (treadmill running for 60 min/day, for 4 weeks). The exercise groups were further divided into subgroups according to training mode: single bout (60 min, daily) vs. multiple bout (three bouts of 20 min, daily). Fasting insulin levels were significantly higher in OLETF than in LETO rats. Among OLETF rats, there were no significant differences in fasting glucose levels between the exercise and the non-exercise groups, but the fasting insulin levels were significantly lower in the exercise group. Body weight and triacylglycerol levels in the liver were significantly higher in OLETF than in LETO rats; however, among OLETF rats, these levels were significantly lower in the exercise than in the non-exercise group. The capillary-to-fiber ratio of the soleus muscle was significantly higher in OLETF than in LETO rats; however, among OLETF rats, the ratio was lower in the exercise group than in the non-exercise group. No significant differences in any of the studied parameters were noted between the single-bout and multiple-bout exercise training modes among either OLETF or LETO rats. These results suggest that low-intensity exercise training improves insulin sensitivity and fatty liver. Additionally, the fact that attenuation of excessive capillarization in the skeletal muscle of OLETF rats was accompanied by improvement in increased body weight.

## Introduction

Exercise improves lipid and glucose metabolism in patients with obesity and type 2 diabetes [1]. The beneficial effect of exercise in obesity and type 2 diabetes is based on the fact that skeletal muscle contraction during exercise increases fatty acid and glucose uptake [2]. Specifically, exercise involves vasodilation and subsequent increase in blood flow to the skeletal muscle [3], which augments the capillary surface area, thereby increasing the accessibility of energy substrates (fatty acids and glucose) to the muscle fibers, with the final outcome of increased fatty acid and glucose uptake.

Vasodilation and increased blood flow to the skeletal muscle are also induced by insulin signaling, independent of exercise. Insulin suppresses hepatic gluconeogenesis and promotes glucose uptake to adipose cells and skeletal muscle fibers, which reduces blood glucose levels; additionally, insulin promotes the phosphorylation of endothelial nitric oxide synthase (eNOS), resulting in vasodilation [4]. Specifically, in the vascular endothelium, which contains insulin receptors, phosphorylated eNOS synthesizes nitric oxide (NO) from L-arginine via the insulin signaling pathway [5]. The produced NO reduces precapillary arteriolar tone, with the resultant vasodilation expanding the number of perfused capillaries in the skeletal muscle [6]. The augmented capillary surface area increases insulin delivery to muscle fibers, promoting glucose uptake.

Obesity and type 2 diabetes are common complications of hyperinsulinemia, a pancreatic response to excessive blood glucose levels promoting glucose uptake by adipose cells and skeletal muscle fibers. Chronic hyperinsulinemia accompanies insulin resistance in the liver, adipose tissue, and skeletal muscle, which are the primary targets of insulin [7]. Insulin resistance is an impairment of insulin-mediated suppression of gluconeogenesis in the liver and of insulin-mediated promotion of glucose uptake in the adipose tissue and skeletal muscle [8]. Insulin resistance is observed not only in metabolically active organs such as the liver, adipose tissue, and skeletal muscle, but also in the vascular endothelium [9]. Vascular insulin resistance diminishes endothelial cell function and impairs insulin-mediated vasodilation [10]. Insulin resistance leads to reduced phosphorylation of eNOS in endothelial cells, which attenuates precapillary arteriolar tone reduction and insulin-induced capillary recruitment [11]. If insulin signaling is impaired in endothelial cells, NO-induced vasodilation and increased blood flow-induced insulin delivery to the skeletal muscle are diminished despite elevated blood insulin levels, resulting in inadequate uptake of glucose by the skeletal muscle [12].

Clinical trials and experimental sutudies have found that exercise can promote glucose uptake and consumption in hyperinsulinemia [13,14,15,16]. For example, Mikus et al. reported that an 8-week program of running improved fasting insulin levels and the insulin sensitivity response during the glucose tolerance test in rats with hyperinsulinemia [17]. Thus, exercise promotes fatty acid and glucose consumption acutely and improves insulin resistance chronically. Long-term exercise is associated with increased expression of numerous genes related to insulin signaling, mitochondrial function, and energy consumption [18]. Additionally, in type 2 diabetes, long-term exercise improves microvascular dysfunction and decreased capillary blood volume in the skeletal muscle, which subsequently augments glucose and insulin delivery as well as glucose uptake [19]. However, Chadderdon et al. reported increased capillary blood volume in rhesus macaques with type 2 diabetes and hyperinsulinemia [20]. It is not clear whether exercise is equally effective when the capillary blood volume is increased.

In a previous study [21], we found increased capillary density in the skeletal muscle of Otsuka Long-Evans Tokushima fatty (OLETF) rats (data not shown). The increased capillary density was found in the soleus muscle, not in the plantaris muscle. In OLETF rats, obesity results from hyperphagia induced by the lack of the satiety signal receptor in the ventromedial nucleus of the hypothalamus [22]. Hyperphagia is also associated with non-alcoholic fatty liver disease, a condition associated with decreased hepatic glucose uptake after eating and increased hepatic gluconeogenesis during fasting [23], which is compensated by hyperinsulinemia [16]. Hyperinsulinemia that compensates for hyperglycemia promotes liver lipid accumulation [24] and inhibits fatty acid release [25], which accelerates obesity in OLETF rats. Although the beneficial effect of exercise on hyperglycemia and hyperinsulinemia in OLETF rats have been known for over two decades [26], the influence of exercise on the blood vessels of skeletal muscles remain unknown.

The purpose of the present study was to investigate the effects of low-intensity exercise training on lipid and glucose metabolism in obesity with hyperinsulinemia, focusing specifically on the capillary density within the skeletal muscle. The American College of Sports Medicine (ACSM) recommends low-intensity exercise training for 20 to 60 min per day to reduce body weight and prevent metabolic diseases [1]. The ACSM guideline states that the daily exercise routine, totaling 20 to 60 min, can be divided into multiple bouts of 10 min or more. Some researchers reported different effects of single-bout and multiple-bout low-intensity exercise training on glucose metabolism and capillary function within the skeletal muscle [19,27,28,29], but these experiments did not involve exercise routines with a comparable total amount of exercise or exercise intensity. Peddie et al. reported different effects on glucose metabolism for single-bout and multiple-bout walking exercise matched in terms of total amount of exercise and exercise intensity [30], but analyzed only blood samples and focused only on the acute effect of exercise. Therefore, in the present study, we also compared the effect of single-bout and multiple-bout exercise training on energy metabolism, with consideration to clinical utility. Specifically, adequate exercise training may help lower health care costs by serving as a complementary therapy to medical treatment for hyperinsulinemia. Our findings may provide biological evidence for the effectiveness of specific exercise prescription in the treatment of individuals with metabolic diseases.

## Materials and methods

### Experimental design

Eighteen-week-old male OLETF rats (n = 11) were used as animal moels of obesity with hyperinsulinemia, while age-matched male Long-Evans Tokushima Otsuka (LETO) rats (n = 12) were used as controls (no obesity, no hyperinsulinemia). The animals were randomly assigned to either non-exercise control groups (OLETF Cont, n = 5; LETO Cont, n = 4)) or exercise groups (OLETF Ex, n = 6; LETO Ex, n = 8). The OLETF Ex and LETO Ex groups were further divided into subgroups according to training mode: single-bout exercise training (OLETF subgroup, n = 3; LETO subgroup, n = 4) vs. multiple-bout exercise training (OLETF subgroup, n = 3; LETO subgroup, n = 4). The animals were housed in a controlled environment with a fixed 12-h light-dark cycle (lights on from 8:00 to 20:00 h) and a constant temperature of 22 ± 2°C. Food and water were provided *ad libitum*. This study was approved by the Institutional Animal Care and Use Committee of Hiroshima University (A16-5) and was performed according to the Hiroshima University Regulations for Animal Experimentation. All experiments were conducted in accordance with the National Institute of Health Guidelines for the Care and Use of Laboratory Animals.

### Low-intensity exercise training protocol

Before randomization, all animals completed a familiarization period of 2 weeks, from 18 to 20 weeks of age, during which they trained starting at a speed of 3 m/min on a 0% gradient, and progressed to 15 m/min on a 5% gradient. At 20 weeks of age, animals in the OLETF Ex and LETO Ex groups started the exercise training program, which involved treadmill running for 60 min/day, 5 days per week, for 4 weeks. Exercise intensity was progressively increased, starting at a speed of 15 m/min on a 5% gradient during the first week, followed by 16 m/min on a 10% gradient during the second week, 17 m/min on a 10% gradient during the third week, and 18 m/min on a 10% gradient during the fourth week. The choice of exercise intensity was based on observations from a previous study involving treadmill running exercise in age-matched OLETF rats [28]. The above-described daily exercise routine was performed as a single bout of 60 min (starting at 9:00 h) by rats in the single-bout exercise subgroups, or as three bouts of 20 min (starting at 9:00, 14:00, and 19:00 h; 60 min/day in total) by rats in the multiple-bout exercise subgroups. The lactate levels in the blood from the lateral caudal vein did not change significantly after exercise (<2.6 mmol/L). The animals in the non-exercise groups (OLETF Cont and LETO Cont) were placed in a different cage, with food and water restriction, to match the intake of rats in the exercise groups (OLETF Ex and LETO Ex, respectively).

### Oral glucose tolerance test

To determine the glucose tolerance relative to body weight, an oral glucose tolerance test (OGTT) was performed at 48 h after the final bout of exercise. The OGTT protocol was based on the approach followed in previous studies [26,31,32]. Specifically, the animals were fasted for 12 h, and glucose (2 g per kg of body weight) was administered via an esophageal feeding tube. Blood samples were obtained from the lateral caudal vein before glucose administration and at 30, 60, and 120 min post-administration. The blood samples were centrifuged at 3000 rpm for 10 min at room temperature and stored at −80°C until measurement of insulin and glucose concentrations. The insulin concentration was measured using a commercially available enzyme-linked immunosorbent assay (ELISA) kit (M1101; Morinaga, Yokohama, Japan), according to the manufacturer’s instructions. Glucose concentrations were measured using a commercially available spectrophotometric assay kit (298–65701; Wako, Osaka, Japan) according to the manufacturer’s instructions. Areas under curve (AUC) were calculate for insulin and glucose levels during OGTT.

### Tissue sampling

At 48 h after the OGTT, the animals were fasted for 12 h and anesthetized by intraperitoneal administration of sodium pentobarbital (50 mg/kg). Once deep anesthesia was established, blood samples were collected from the caudal vena cava and the liver, epididymal adipose tissue, and soleus muscle were immediately removed, weighed, frozen in liquid nitrogen, and stored at -80°C until further analysis.

### Analysis of the blood

The blood samples collected from the caudal vena cava were centrifuged at 3000 rpm for 10 min at room temperature, and the plasma fraction was stored at -80°C until measurement of free fatty acid (FFA), triacylglycerol (TAG), tumor necrosis factor-α (TNF-α), and adiponectin concentrations. FFA and TAG concentrations were measured using a commercially available spectrophotometric assay kit (294–63601 and 290–63701, respectively; both from Wako), whereas TNF-α and adiponectin concentrations were measured using a commercially available ELISA kit (865.000.096 from Diaclone, Besancon, France; ERA2500-1 from Assaypro, St Charles, MO, USA; respectively) according to the manufacturer’s instructions.

### Analysis of the liver

Using a cryostat, transverse sections with a thickness of 10 μm were obtained from samples of the left lateral lobe of the liver and mounted on amino-silane-coated glass slides. The sections were stained with Sudan red for histological evaluation. The TAG concentration in the liver was determined according to the method described by Folch et al. [33]. Briefly, the frozen samples from the left lateral lobe of the liver were washed with saline and homogenized with chloroform/methanol mixture (2:1, v/v). The homogenates were incubated for 60 min at room temperature, filtrated, and centrifuged at 3000 rpm for 10 min at room temperature. After removing the aqueous phase, the chloroform phase was collected and evaporated by drying. The obtained fats were dissolved in isopropanol, and TAG concentration was measured using spectrophotometry.

### Analysis of the epididymal adipose tissue

Transverse sections with a thickness of 30 μm were obtained from the epididymal adipose tissue, following the same protocol as that used for obtaining the liver tissue sections. The sections were stained with Sudan red. Using dedicated software (ImageJ; NIH, Bethesda, MD, USA), the diameter of adipose cells was measured in two randomly chosen fields. At least 100 adipose cells were measured per animal.

### Analysis of the soleus muscle

Using a cryostat, transverse sections with a thickness of 10 μm were obtained from tissue samples taken from the middle part of the soleus muscle. The sections were mounted on amino-silane-coated glass slides and stained with Sudan red for histlogic eveluation of lipid localization. Such sections were also stained for myofibrillar adenosine triphosphatase (ATPase) activity to assess the composition and characteristics of muscle fibers. Specifically, the sections were preincubated in barbital-acetate buffer (pH 4.2) for 5 min at room temperature and then incubated in 0.1 M barbital buffer containing 0.18 M CaCl_2_ and 4 mM ATP (pH 9.4) for 45 min at room temperature. After incubation, the sections were washed with 1% CaCl_2_ and 2% CoCl_2_ every 3 min. After washing with 0.01 M sodium barbital, the sections were stained with 1% ammonium sulfide. The sections stained with ATPase were used to determine the composition of muscle fiber types and to measure the cross-sectional area of muscle fibers of each type. When evaluating muscle fiber type distribution and cross-sectional area, all muscle fibers noted in a cross-section were used (at least 700 muscle fibers per animal). Finally, such sections were also stained for alkaline phosphatase activity to visualize the capillaries within the skeletal muscle. Specifically, the sections were incubated for 45 min at 37°C in 0.1% 5-bromo-4-chloro-3-indolyl phosphate p-toluidine salt and 0.1% nitro blue tetrazolium in 0.2 M borate buffer. The sections stained for alkaline phosphatase activity were used to determine the capillary-to-fiber (C/F) ratio. For the measurement of C/F ratio, three random fields were chosen, and >100 muscle fibers were measured per animal. The quantifications were performed using ImageJ (NIH). Additionally, the relationship between the C/F ratio and body weight was investigated as a supplementary experiment. Seventeen-week-old male OLETF rats (n = 5, body weight 568 ± 23 g) and body weight-matched male LETO rats (n = 5, body weight 551 ± 26 g, 40-week-old) were used to determine whether the increased C/F ratio in the skeletal muscle of OLETF rats is due to increased body weight.

Frozen muscle samples were homogenized in ice-cold homogenizing buffer containing 50 mM Tris-HCl (pH 7.8), 0.15 M NaCl, and 1% protease inhibitor cocktail (25955–11; Nacalai tesque, Kyoto, Japan). The homogenates were centrifuged at 18000 rpm for 30 min at 4°C. Following the measurement of total the protein concentration using a protein determination kit (500–0006; Bio-Rad Laboratories, Hercules, CA, USA), NOS activity was measured using a commercially available assay kit (NB78; Oxford Biomedical Research, Rochester Hills, MI, USA) according to the manufacturer’s instructions. Additionally, citrate synthase (CS) activity was measured by spectrophotometry. The muscle samples were homogenized in homogenizing buffer containing 10 mM 4-(2-hydroxyethyl)-1-piperazineethanesulfonic acid (pH 7.3), 11.5% sucrose, 0.1% Triton X-100, 1 mM dithiothreitol, and 5% protease inhibitor cocktail (25955–11; Nacalai Tesque, Kyoto, Japan). After centrifugation at 1,500*g* for 10 min at 4°C, the supernatants were diluted 10-fold in distilled water. The diluted homogenate was added to a cuvette containing a reaction mix of 1 mM 5,5′-dithiobis and 10 mM acetyl-coenzyme A. After incubation for 10 min at 25°C, 10 mM oxaloacetate was added to the cuvette, and the optical density of the reaction solution was measured at a wavelength of 412 nm for 3 min. CS activity was calculated assuming an extinction coefficient of 1290 L·mol^-1^·cm^-1^.

Total RNA was isolated using TRIzol reagent (15596–026; Invitrogen, Tokyo, Japan). Reverse transcription was performed using the High-Capacity cDNA Reverse Transcription Kit (4374966; Applied Biosystems, Foster City, CA, USA), and the resultant cDNA samples were stored at −20°C until analysis. The expression levels of vascular endothelial growth factor (VEGF; Rn01511602_m1) and thrombospondin-1 (TSP-1; Rn01513693_m1) mRNA were quantified using quantitative polymerase chain reaction (qPCR) with TaqMan Gene Expression Assays (Applied Biosystems). The relative expression levels of VEGF and TSP-1 were inferred by normalizing the quantity of cDNA template for each gene against the quantity of cDNA for the normalization gene 18S (Rn03928990_g1). The cDNA concentration at each qPCR cycle was plotted to obtain a standard curve, and the relative gene expression was obtained as the value at the threshold line. qPCR was performed using the PCR Fast Advanced Master Mix (Applied Biosystems) in a 96-well reaction plate. Each well contained cDNA template, PCR Fast Advanced Master Mix polymerase kit, and TaqMan Gene Expression Assays with the corresponding primers and probe. All samples and non-template control reactions were performed in CFX96 (Bio-Rad Laboratories) under the following conditions: 50°C for 2 min and 95°C for 20 s, followed by 40 cycles at 95°C for 3 s and 60°C for 30 s.

### Statistical analysis

Data were expressed as means ± standard deviation. The main effect of animal strain, exercise, and interaction between animal strain and exercise were evaluated using two-way analysis of variance (ANOVA). The significance of differences between subgroups defined in terms of exercise mode (i.e., single-bout vs. multiple-bout exercise training) was evaluated using one-way ANOVA, followed by Tukey’s honest significant difference post hoc test. The significance of differences in body weight between the start and end of the experiment was evaluated using the paired t-test. The significance of differences between weight-matched OLETF and LETO rats was evaluated using the student t-test. Statistical significance was set at *p* < 0.05.

## Results

### Changes in body weight

**Fig 1:**
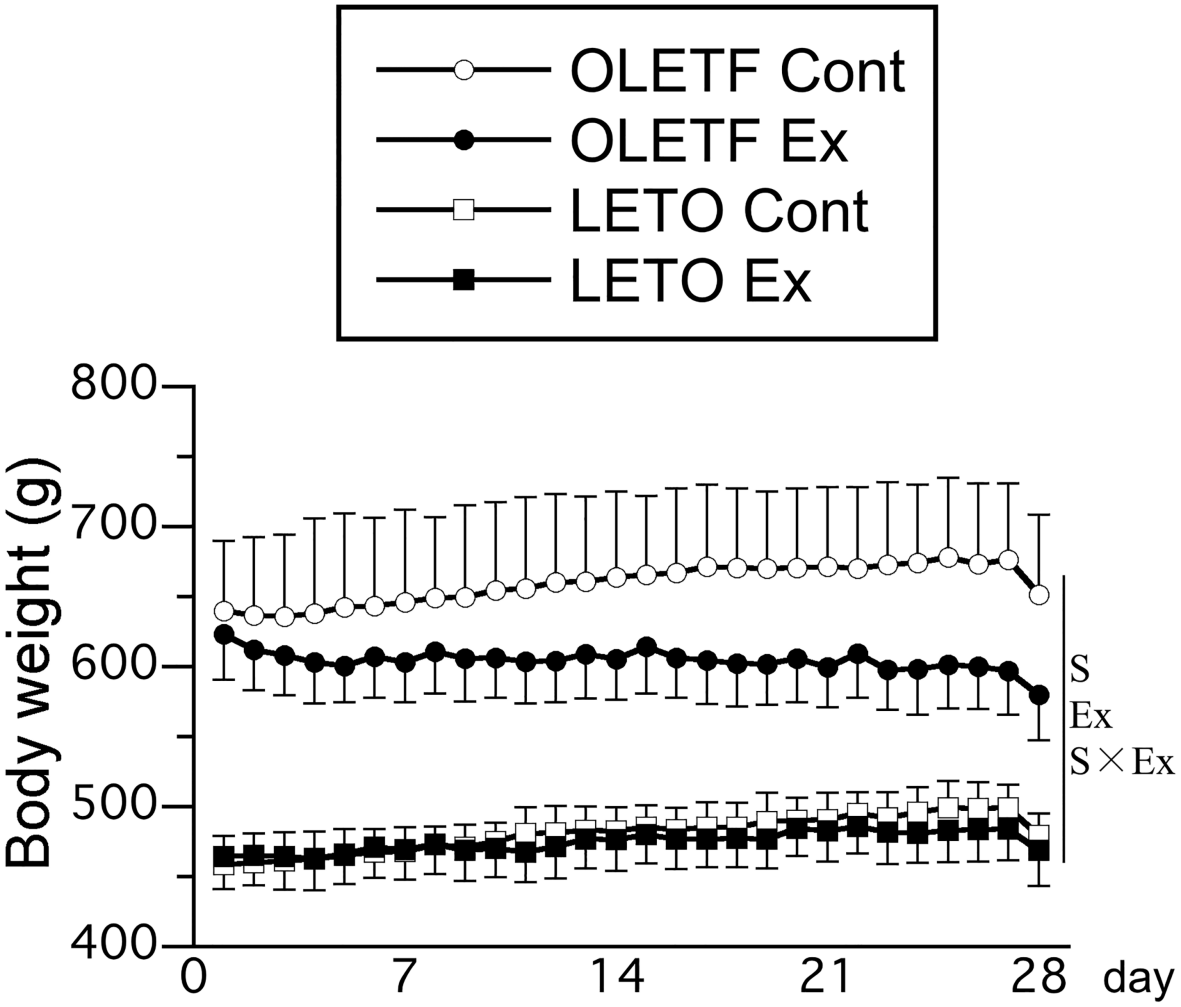
Time course of body weight changes after exercise. Values represent means ± standard deviation. S, Ex, and S × Ex denote the main effects of animal strain, exercise, and interaction between animal strain and exercise, respectively. These effects were significant at 28 days (*p* < 0.05).

Throughout the experimental period, body weight was significantly higher in OLETF rats than in LETO rats (Fig 1). In the OLETF Cont group, body weight was slightly higher at the end of experiment than at the start of experiment (start, 639 ± 50 g; end, 651 ± 57 g; *p* = 0.33). In contrast, in the OLETF Ex group, body weight was significantly lower at the end of experiment than at the start of experiment (start, 623 ± 32 g; end, 580 ± 32 g; *p* < 0.01). Body weight was lower in rats that exercised than in those that did not, and the main effect of exercise on body weight was significant after 16 days of intervention with exercise training. The interaction between animal strain and exercise was significant at 18, 19, 21, 23, 24, 25, 27, and 28 days. Finally, no significant differences in body weight were noted between the single-bout and multiple-bout exercise subgroups of either the OLETF Ex or LETO Ex group (subgroup data not shown independently; for each exercise group, pooled data from the single-bout and multiple-bout exercise subgroups are shown).

### Insulin and glucose levels during OGTT

**Fig 2:**
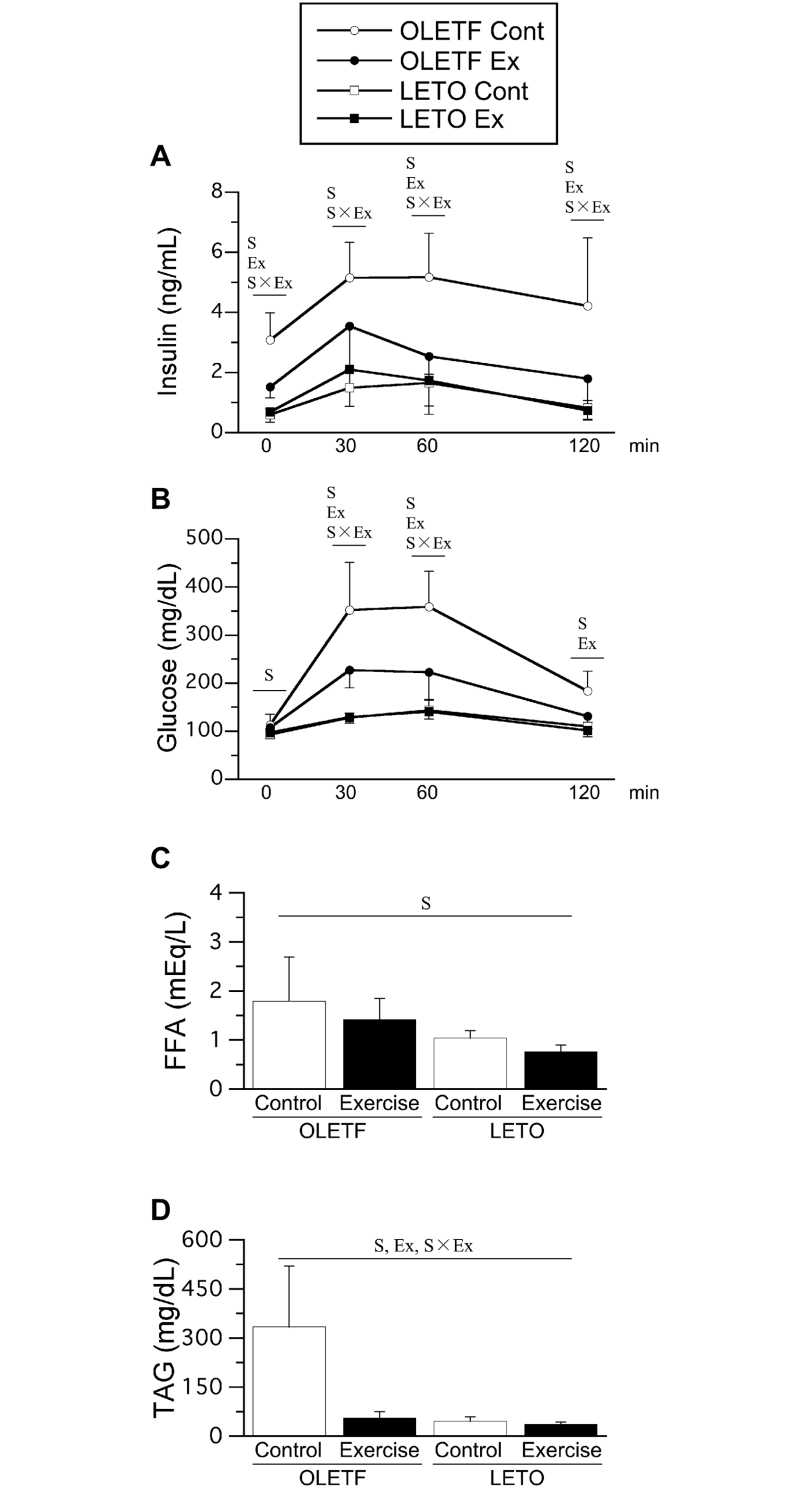
Insulin levels (A) and glucose levels (B) during OGTT, fasting plasma FFA levels (C), and TAG levels (D). Time zero means fasting. Values represent means ± standard deviation. S, Ex, and S × Ex denote significant (*p* < 0.05) main effects of animal strain, exercise, and interaction between animal strain and exercise, respectively.

Fasting insulin level was significantly higher in OLETF rats than in LETO rats (Fig 2A). However, the fasting insulin level was significantly lower in the OLETF Ex group than in the OLETF Cont group. No significant differences in fasting insulin levels were noted between the LETO Cont and LETO Ex groups. Similarly, no significant differences in fasting insulin levels were noted between the single-bout and multiple-bout exercise subgroups of either the OLETF Ex or LETO Ex group (only combined data shown). In all groups, glucose administration led to an obvious increase in insulin levels. Although the insulin levels decreased at 60 and 120 min after glucose administration, the rate of decline was slow; the insulin levels remained elevated for a long time only in the OLETF Cont group. The mean value of AUC for insulin level was 190 ± 64 in the OLETF Cont group, 127 ± 126 in the OLETF Ex group, 81 ± 24 in the LETO Cont group, and 91 ± 62 in the LETO Ex group. The main effect of animal strain on AUC for insulin level was significant, as AUC for insulin level were significantly higher in OLETF rats than in LETO rats. The main effect of exercise and the interaction between animal strain and exercise were insignificant on AUC for insulin level. No significant differences in AUC for insulin level were noted between the single-bout and multiple-bout exercise subgroups of either the OLETF Ex or LETO Ex group.

Fasting glucose levels were significantly higher in OLETF rats than in LETO rats (Fig 2B). Following glucose administration, glucose levels increased gradually in all groups. Elevated glucose levels persisted for a long time in the OLETF Cont group. Glucose levels were significantly higher in OLETF rats than in LETO rats at 30, 60, and 120 min after glucose administration. However, glucose levels were significantly lower in the OLETF Ex group than in the OLETF Cont group. No significant differences in glucose levels were noted between the LETO Cont and LETO Ex groups. The mean value of AUC for glucose level was 20390 ± 5346 in the OLETF Cont group, 9505 ± 4503 in the OLETF Ex group, 3827 ± 469 in the LETO Cont group, and 4122 ± 2916 in the LETO Ex group. The main effects of animal strain and exercise, and the interaction between animal strain and exercise on AUC for insulin level were significant, as AUC for glucose level were significantly higher in OLETF rats than in LETO rats. However, AUC for glucose level were significantly lower in the OLETF Ex group than in the OLETF Cont group. Additionally, no significant differences in glucose levels were noted between the single-bout and multiple-bout exercise subgroups of either the OLETF Ex or LETO Ex group.

### Plasma FFA and TAG levels

The main effect of animal strain on fasting plasma FFA levels was significant, as fasting plasma FFA levels were significantly higher in OLETF rats than in LETO rats (Fig 2C). Fasting plasma TAG levels were also significantly higher in OLETF rats than in LETO rats (Fig 2D). Fasting plasma TAG levels were significantly lower in the OLETF Ex group than in the OLETF Cont group, while no significant differences were noted between the LETO Cont and LETO Ex groups. Additionally, no significant differences in fasting plasma TAG levels were noted between the single-bout and multiple-bout exercise subgroups of either the OLETF Ex or LETO Ex group (only combined data shown).

### Plasma TNF-α and adiponectin levels

In the blood samples collected from the vena cava, TNF-α expression levels were below the minimum detectable threshold (31.25 pg/mL) for among all groups. The mean adiponectin levels were 10779 ± 3628 ng/mL in the OLETF Cont group, 8247 ± 1667 ng/mL in the OLETF Ex group, 5392 ± 968 ng/mL in the LETO Cont group, and 5239 ±727 ng/mL in the LETO Ex group. Adiponectin levels were significantly higher in OLETF rats than in LETO rats. No significant differences were noted between the OLETF Cont and OLETF Ex groups, or between the LETO Cont and LETO Ex groups.

### Liver

**Fig 3:**
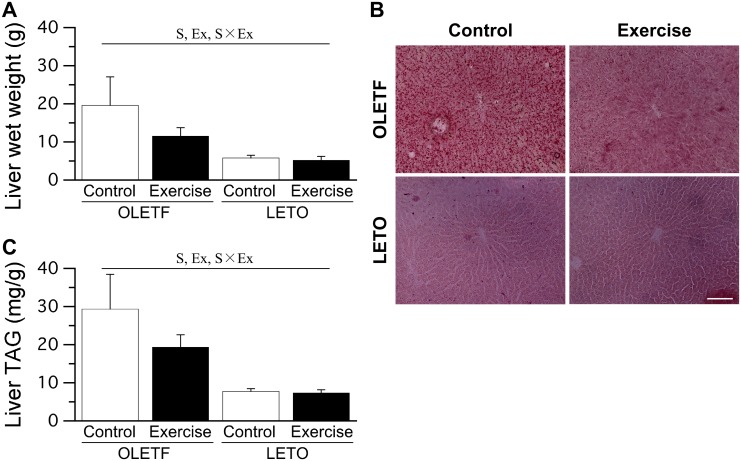
Liver wet weight (A), representative liver sections stained with Sudan red (B), and liver TAG levels (C). Bar = 100 μm. Values represent means ± standard deviation. S, Ex, and S × Ex denote significant (*p* < 0.05) main effects of animal strain, exercise, and interaction between animal strain and exercise, respectively.

The wet weight of the liver was significantly higher in OLETF rats than in LETO rats (Fig 3A). Liver wet weight was significantly lower in the OLETF Ex group than in the OLETF Cont group. No significant differences in liver wet weight were noted between the LETO Cont and LETO Ex groups. Additionally, no significant differences in liver wet weight were noted between the single-bout and multiple-bout exercise subgroups of either the OLETF Ex or LETO Ex group (only combined data shown).

Ectopic lipid accumulation in the liver was clearly observed in OLETF rats (Fig 3B). Liver TAG levels were significantly higher in OLETF rats than in LETO rats (Fig 3C). However, liver TAG levels were significantly lower in the OLETF Ex group than in the OLETF Cont group. No significant differences in liver TAG levels were noted between the LETO Cont and LETO Ex groups. Additionally, no significant differences in liver TAG levels were noted between the single-bout and multiple-bout exercise subgroups of either the OLETF Ex or LETO Ex group (only combined data shown).

### Epididymal adipose tissue

**Fig 4:**
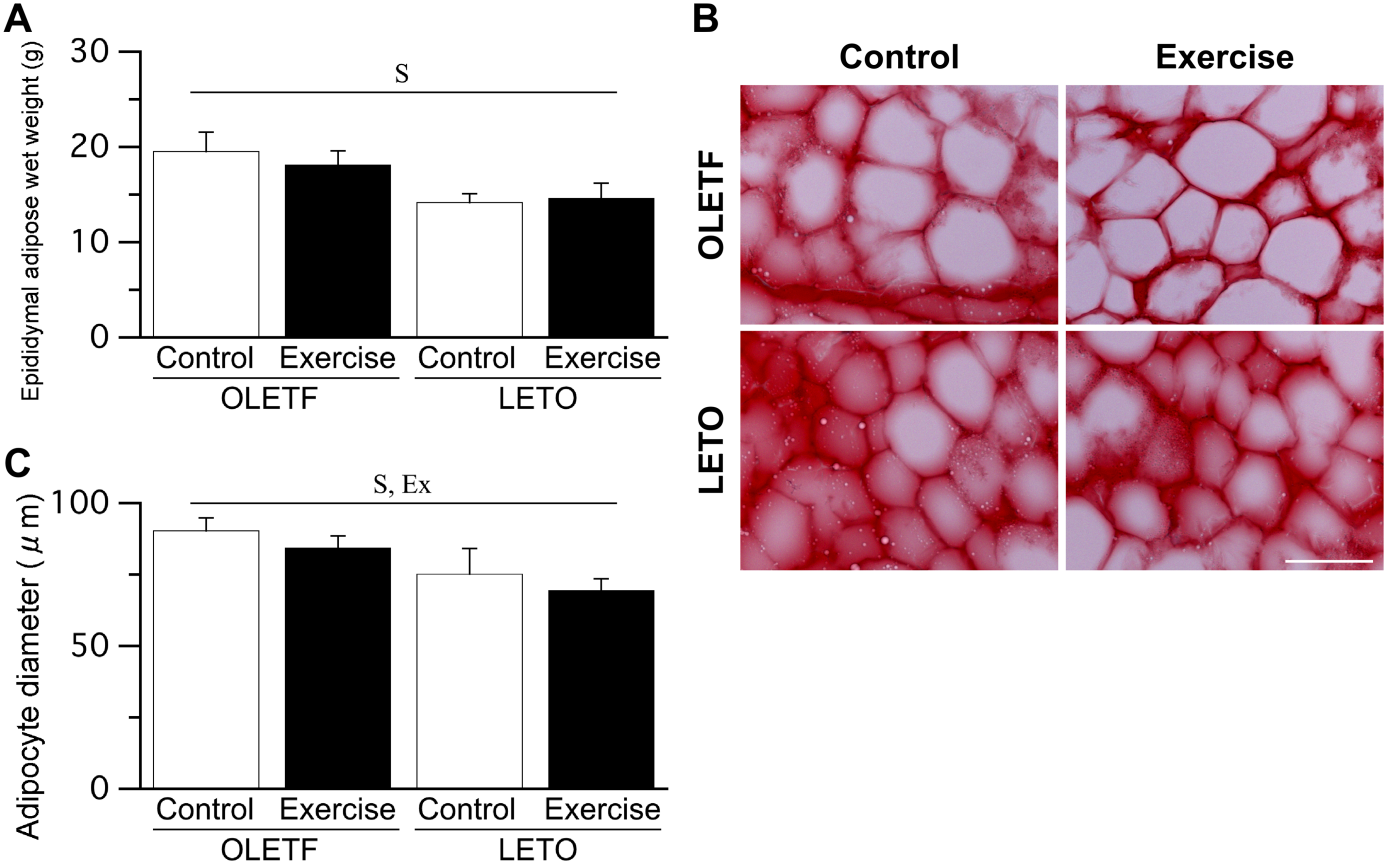
Epididymal adipose wet weight (A), representative epididymal adipose sections stained with Sudan red (B), and adipocyte diameter (C). Bar = 100 μm. Values represent means ± standard deviation. S, Ex, and S × Ex denote significant (*p* < 0.05) main effects of animal strain, exercise, and interaction between animal strain and exercise, respectively.

The wet weight of epididymal adipose tissue was significantly higher in OLETF rats than in LETO rats (Fig 4A). Although adipocyte enlargement was more frequently observed in OLETF rats than in LETO rats, hypertrophic adipocytes with a diameter >130 μm were rare in both OLETF and LETO rats (Fig 4B). The adipocyte diameter was significantly larger in OLETF rats than in LETO rats (Fig 4C). The main effect of exercise on adipocyte diameter was significant, as adipocyte diameter was significantly lower in rats that exercised than in those that did not. No significant differences in the wet weight of epididymal adipose tissue and adipocyte diameter were noted between the single-bout and multiple-bout exercise subgroups of either the OLETF Ex or LETO Ex group (only combined data shown).

### Soleus muscle

**Fig 5:**
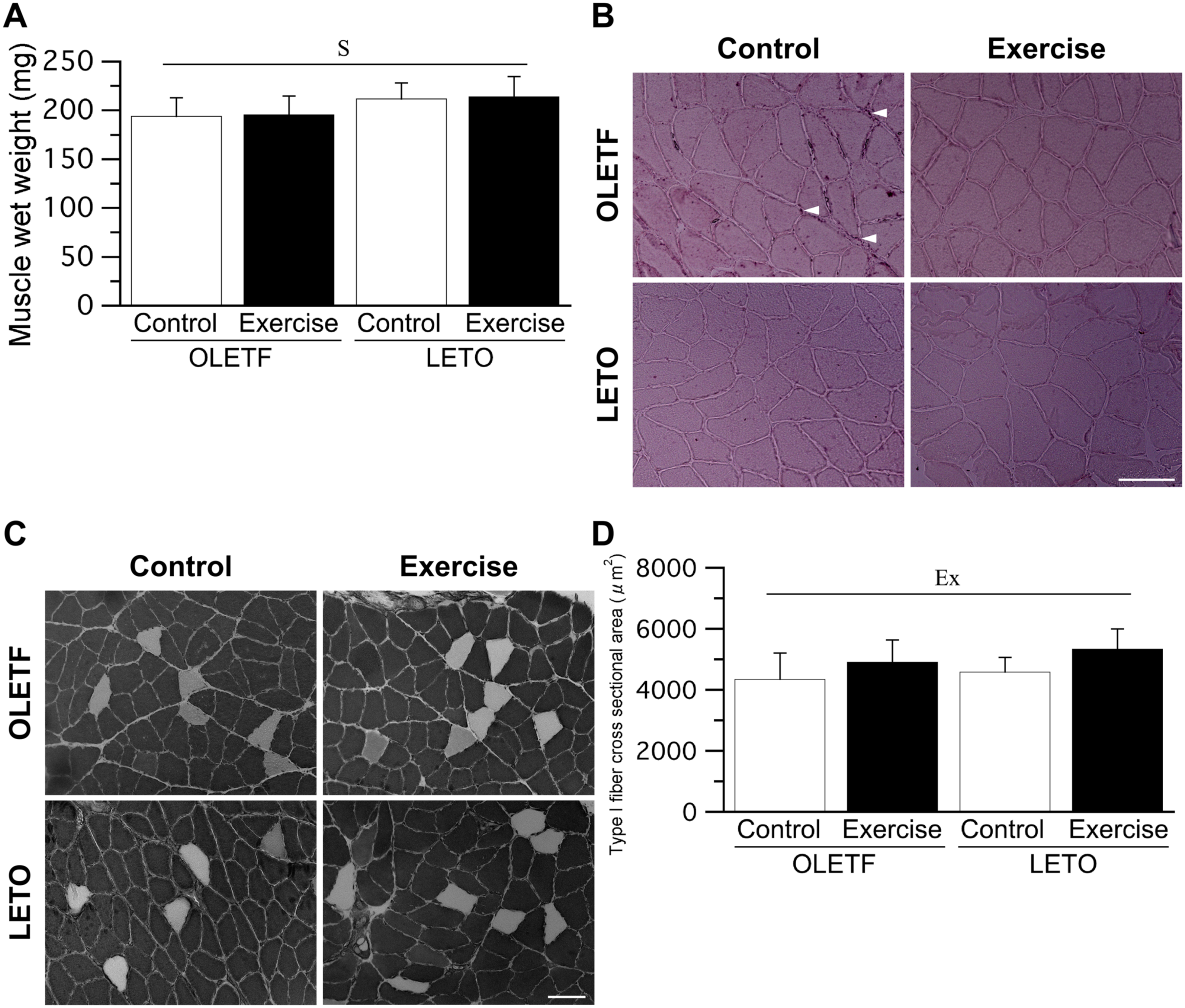
Muscle wet weight (A), representative sections of the soleus muscle stained with Sudan red (B) and staind for ATPase activity (C), muscle fiber type distribution (D), and muscle fiber cross-sectional area (E). Arrow heads: ectopic lipid accumulation. Bar = 100 μm. Values represent means ± standard deviation., Ex, and S × Ex denote significant (*p* < 0.05) main effects of animal strain, exercise, and interaction between animal strain and exercise, respectively.

The main effect of animal strain on soleus muscle wet weight was significant, as soleus muscle wet weight was significantly lower in OLETF rats than in LETO rats (Fig 5A). Ectopic lipid accumulation was observed around the sarcolemma, especially in OLETF rats (Fig 5B). However, lipid droplets within the muscle fiber were not observed in either OLETF or LETO rats. ATPase staining revealed that the soleus muscles were composed of type I and IIA fibers (Fig 5C), with type I fiber dominant in all groups. The mean proportion of type I fiber was 95 ± 6% in the OLETF Cont group, 99 ± 1% in the OLETF Ex group, 96 ± 2% in the LETO Cont group, and 96 ± 3% in the LETO Ex group. No significant differences in muscle fiber type distribution were observed among all groups. The mean value of CS activity was 3.1 ± 1.7 U·min^-1^·g^-1^ in the OLETF Cont group, 4.1 ± 1.4 U·min^-1^·g^-1^ in the OLETF Ex group, 4.2 ± 1.0 U·min^-1^·g^-1^ in the LETO Cont group, and 3.4 ± 1.2 U·min^-1^·g^-1^ in the LETO Ex group. No significant differences in CS activity were noted among the groups. The main effect of exercise on the cross-sectional area of type I fiber was significant, as the cross-sectional area of type I fiber was significantly higher in rats that exercised than in those that did not (Fig 5D).

**Fig 6:**
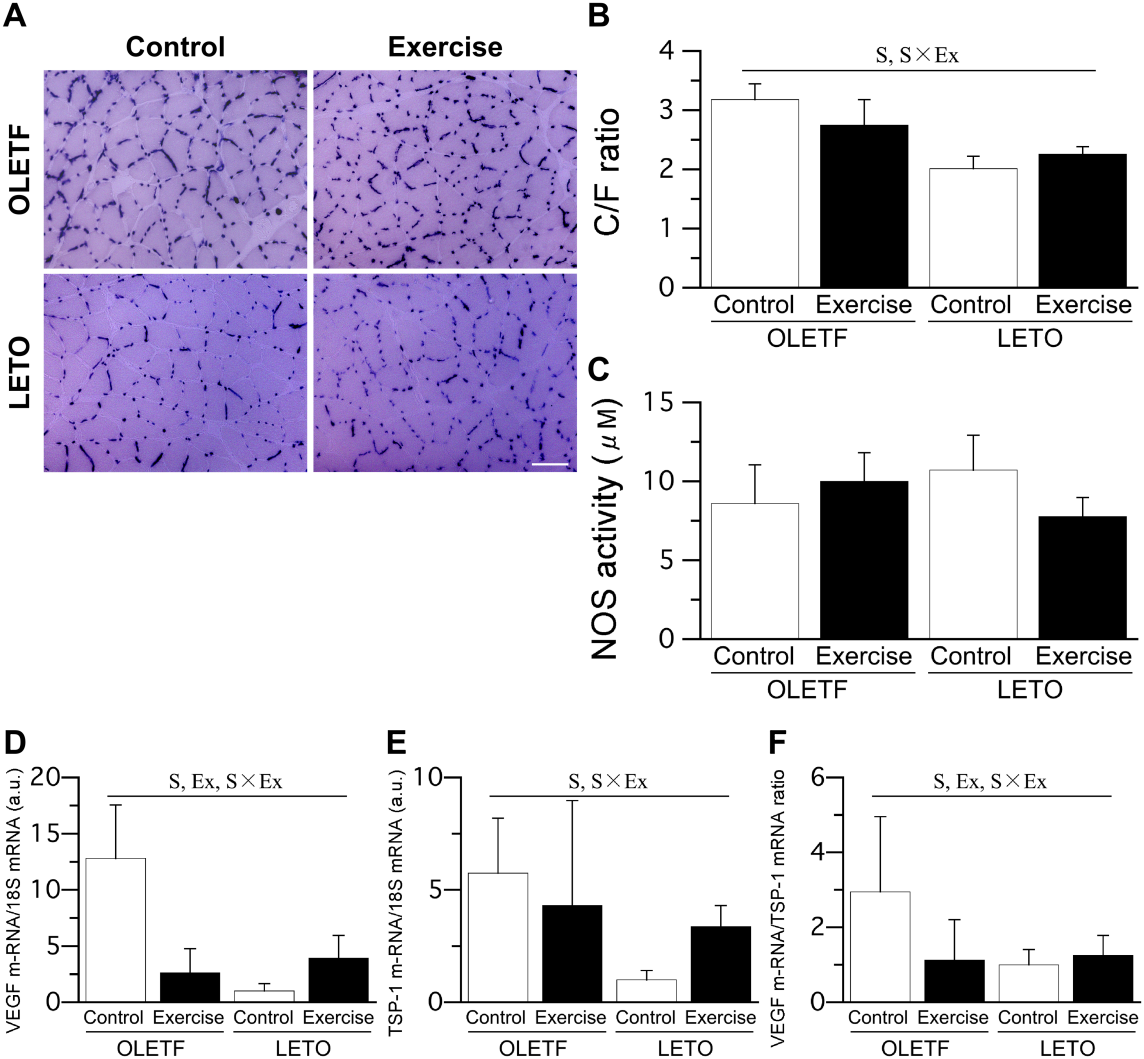
Representative sections stained with alkaline phosphatase (A), C/F ratio (B), NOS activity (C), expression levels of VEGF mRNA (D) and TSP-1 (E) mRNA, and ratio of VEGF to TSP-1 mRNA expression level (F) in the soleus muscle. Bar = 100 μm. For the expression levels of VEGF and TSP-1 mRNA, the values were calculated as fold changes relative to the LETO Cont group. Values represent means ± standard deviation. S, Ex, and S × Ex denote significant (*p* < 0.05) main effects of animal strain, exercise, and interaction between animal strain and exercise, respectively.

Staining for alkaline phosphatase activity revealed pronounced capillarization in the soleus muscle of OLETF rats (Fig 6A). The C/F ratio was significantly higher in OLETF rats than in LETO rats (Fig 6B). However, the C/F ratio was significantly lower in the OLETF Ex group than in the OLETF Cont group. No significant differences regarding NOS activity in the soleus muscle were noted among the groups (Fig 6C). However, VEGF mRNA expression levels were significantly higher in OLETF rats than in LETO rats, and significantly lower in the OLETF Ex group than in the OLETF Cont group (Fig 6D). The expression levels of TSP-1 mRNA were significantly higher in OLETF rats than in LETO rats (Fig 6E). The ratio of VEGF to TSP-1 mRNA expression levels, which reflects the relative expression of angiogenic and anti-angiogenic factors, was significantly higher in OLETF rats than in LETO rats, but significantly lower in the OLETF Ex group than in the OLETF Cont group (Fig 6F). Finally, no significant differences regarding capillary density or angiogenesis in the skeletal muscle were noted between the single-bout and multiple-bout exercise subgroups of either the OLETF Ex or LETO Ex group.

### The analyses in body weight-matched OLETF and LETO rats

No significant differences in the C/F ratios were noted between OLETF and LETO rats (Table 1). The soleus muscle wet weight in OLETF rats was significantly lower than that in LETO rats.

**Table 1:** C/F ratio in body weight-matched OLETF and LETO rats.

	OLETF	LETO	*P* value
Body weight, g	568 ± 23	551 ± 26	0.30
C/F ratio	2.72 ± 0.48	2.86 ± 0.29	0.61
Fasting insulin, ng/mL	1.90 ± 1.00	0.80 ± 0.28	0.04
Fasting glucose, mg/dL	108 ± 7	111 ± 19	0.77
TAG, mg/dL	65 ± 22	41 ± 10	0.05
Muscle wet weight, mg	191 ± 18	240 ± 13	< 0.01

Values represent means ± standard deviation.

## Discussion

Although the fasting glucose levels did not differ between the OLETF Cont and OLETF Ex groups, fasting hyperinsulinemia was milder in the OLETF Ex group. These results indicate that glucose levels were kept in the OLETF Ex group even though insulin secretions were low. Following glucose administration based on body weight, the insulin levels were lower in the OLETF Ex group than in the OLETF Cont group. These results indicate that insulin secretions required for glucose intake was low in the OLETF Ex group. Impaired insulin sensitivity is induced by obesity [34]; additionally, higher TNF-α and FFA levels and lower adiponectin levels are identified as major triggers for obesity-induced insulin resistance [35,36]. In our study, OLETF rats had slightly higher FFA levels but no abnormalities in TNF-α or adiponectin levels, indicating that the excessive insulin secretion observed in the OLETF Cont group must be early disorder in insulin sensitivity. Our findings indicate that repeated exercise training (i.e., in the OLETF Ex group) was effective in attenuating the severity of the disorder by promoting glucose consumption. In obesity, vascular insulin resistance develops early and its onset precedes insulin resistance in the liver, adipose tissue, and skeletal muscle [37]. Chadderdon et al. reported that capillary blood volume in the skeletal muscle increases consistently in the early stage of obesity-induced vascular insulin resistance, and subsequently falls abruptly with the establishment of pancreatic secretory dysfunction [20]. In the present study, OLETF rats showed increased capillary density in the skeletal muscle in the absence of insulin hyposecretion. Assuming that higher capillarization in the skeletal muscle reflects an increased capillary blood volume, we may conclude that the OLETF rats used in the present study had vascular insulin resistance in an early stage. The fact that attenuation of excessive capillarization in the skeletal muscle of OLETF rats was accompanied by improvement in increased body weight.

We found that low-intensity exercise training accelerated TAG consumption in OLETF rats, which is in agreement with previous findings reported by Rector et al. [38,39]. During aerobic exercise, the skeletal muscle consumes fatty acids as the main energy substrate [40]. We found no difference between pre-exercise and post-exercise lactate levels. Interestingly, we found that exercise training was associated with a slight decrease in adipocyte diameter but no decrease in plasma FFA levels or adipose mass in OLETF rats. Insulin secretion is suppressed during aerobic exercise, which stimulates fatty acid release from the adipose tissue to maintain sufficient levels of energy substrate [25]. In OLETF rats, exercise-induced fatty acid release from the adipose tissue may be suppressed as a result of hyperinsulinemia; reduced fatty acid release may inhibit plasma FFA consumption, which could explain our present findings. In contrast with the results of the present study, Morris et al. reported that voluntary exercise on the running wheel is associated with reduced volume of epididymal adipose tissue in OLETF rats [41]. Similarly, Linden et al. reported that a 12-week program of treadmill running exercise is associated with reduced FFA levels in 32-week-old OLETF rats [42]. The discrepancy between our present results and those of previous studies could be due to the form of exercise (i.e., voluntary exercise vs. treadmill running exercise), exercise period (i.e., 12 weeks vs. 4 weeks), and disease stage (i.e., in 32-week-old vs. 24-week-old rats). We found that, in contrast to the plasma FFA levels and adipose mass, which did not appear to be affected by exercise, TAG levels in both the plasma and liver decreased drastically in association with exercise training in OLETF rats. These findings suggest that the skeletal muscle in OLETF rats may use TAG as the main energy substrate during exercise, and the capillarization in the skeletal muscle of OLETF rats may enhance TAG consumption during low-intensity exercise training. Lipoprotein lipase (LPL) locates on the luminal surface of endothelial cells in the capillaries of the skeletal muscle [43]. LPL hydrolyzes bloodstream TAG into three FFAs and glycerol. Because LPL activity correlates positively with capillary density in the skeletal muscle [43], and since we found that the C/F ratio was higher in OLETF rats than in LETO rats, it is likely that LPL activity is higher in OLETF rats than in LETO rats. Higher LPL activity in the skeletal muscle may potentiate the effect of exercise-induced increase in blood flow in terms of the improvement of hypertriacylglycerolemia and subsequent fatty liver in OLETF rats.

The capillary density in the soleus muscle was significantly higher in OLETF rats than in LETO rats. Increased capillary density in the skeletal muscle is usually accompanied by muscular hypertrophy [44] and a shift to slow muscle [45]. However, in the present study, no significant difference in muscle fiber cross-sectional area or muscle fiber type distribution was noted between OLETF and LETO rats. Although we had expected that an increase in capillary density would be accompanied by an increase in NOS activity, we found similar NOS activity in OLETF and LETO rats. Moreover, exercise training was associated with decreased ratio of VEGF to TSP-1 mRNA expression levels and capillary density in OLETF rats, without any changes in NOS activity. Bender et al. reported that phosphorylated eNOS levels are reduced in isolated feed arteries from the gastrocnemius muscle of OLETF rats [46]. Therefore, we speculate that the capillarization in the skeletal muscle of OLETF rats is an unusual adaptation, as is the case with muscular hypertrophy and a shift to slow muscle characteristics; specifically, the increase in the number of capillaries serves to compensates for endothelial dysfunction and impaired vasodilation. Martin et al. reported that endurance exercise enhances endothelium-dependent dilation in response to acetylcholine in second-order arterioles perfusing the gastrocnemius muscle of OLETF rats [28]. Mikus et al. reported that daily wheel running prevents the decline in insulin-stimulated vasodilation in second-order arterioles perfusing the gastrocnemius muscle of OLETF rats through NO-mediated vasoreactivity [47]. It is possible that, in the present study, exercise training enhanced endothelial function in response to insulin and reduced the progression of capillarization, although it is not possible to verify such a conclusion based on the data we collected. In contrast to the present findings, previous observations suggest that muscle fiber cross-sectional area [48] and distribution of slow muscle fiber [21] are decreased in type 2 diabetes. Additionally, the previous studies reported that the C/F ratio of skeletal muscle did not change [49] or decrease in obesity [50] and type 2 diabetes with hyperinsulinemia [51]. Gavin et al. reported that obese patients with mild hyperglycemia and severe hyperinsulinemia did not demonstrate any changes in the C/F ratio [52]. The C/F ratio of the skeletal muscle is still unreported in OLETF rats. However, the subjects in the study by Gavin et al. had similar on metabolic characteristics to OLETF rats in the present study. Although the underlying factors of an increased C/F ratio are uncertain in OLETF rats, some factors such as decreased VEGF, increased TSP-1 [53], and increased corticosterone [54,55] are thought to be associated with a decreased C/F ratio. In the analyses using body weight-matched OLETF and LETO rats, no significant differences in the C/F ratio were noted between OLETF and LETO rats. These results implied that the values of the C/F ratio involve body weight rather than muscle mass. We did not identify the underlying factors of an increased C/F ratio in OLETF rats. However, because exercise training decreased both the C/F ratio and body weight in OLETF rats, excessive body weight could possibly be one of the factors for the increased C/F ratio.

We found that, although exercise improved hyperinsulinemia and fatty liver in OLETF rats there were no significant differences in the effects of single-bout and multiple-bout exercise training. Peddie et al. reported that multiple bouts of physical activity are more effective than a single bout for improving postprandial hyperglycemia in healthy humans [30]. Their study enrolled healthy human subjects that regularly consumed experimental food every 3 h. In our study, OLETF and LETO rats were used; these experimental rodents were provided with food and water *ad libitum* throughout the entire light-dark cycle. The discrepancy between our results and those of Peddie et al. may be related to differences in food intake patterns and thus in circadian variation of blood glucose levels. In individuals with elevated glucose level, even a small amount of exercise performed after every food intake could be effective provided that a regular food intake pattern is established. We had expected that a single bout of exercise would be more effective than multiple bouts in terms of lipid metabolism. Specifically, a small amount of time is required to achieve vasodilation and complete lipid utilization in the skeletal muscle during exercise [3]. Therefore, compared to multiple but shorter bouts of exercise, a single continuous bout of exercise is more likely to achieve complete lipid utilization in the muscle, and may be more effective because exercise would be continued for longer after complete lipid utilization. However, we found no difference in lipid metabolism between single-bout and multiple-bout exercise training in OLETF rats, which could be attributed to hyperinsulinemia-induced suppression of fatty acid release. In hyperinsulinemia, the skeletal muscle may rely on any energy substrate (except FFA from the adipose tissue) during exercise, such as intramyocellular TAG. Depleted intramyocellular TAG is replenished by TAG from plasma after several hours [43]. Therefore, the decreased plasma and liver TAG in the OLETF Ex group may reflect a mechanism of compensation for the depletion of intramyocellular TAG during exercise. However, in this study, the blood samples were obtained only after 96 h from the last exercise, and the number of sample was small; thus, the assumption that energy consumption depends on the total amount of exercise should be treated with caution.

To summarize, we found that low-intensity exercise training improves hyperinsulinemia, hypertriacylglycerolemia, and fatty liver, with no significant differences in the effects of a single bout and of multiple bouts of exercise. Additionally, we found capillarization in the skeletal muscle of rats with obesity and hyperinsulinemia, but low-intensity exercise training was associated with changes in capillarization, which were accompanied with improvements in increased body weight and fatty liver. This study demonstrated the effectiveness of exercise in obesity with hyperinsulinemia. Nevertheless, some limitations should be noted. Laughlin et al. reported that, in OLETF rats, exercise-induced changes in arteriolar gene expression patterns differ with muscle fiber type composition and along the arteriolar tree [56]. However, we did not harvest arterioles or vascular tissue, and all analyses pertaining to the blood vessels of the skeletal muscle were performed using homogenates of the soleus muscle tissue. Furthermore, we did not measure capillary vasodilation function or blood volume, insulin resistance in each harvested tissue, and amount of intramyocellular TAG. Finally, the number of animals was limited, especially in the single-bout and multiple-bout exercise subgroups. If significant differences in fasting insulin level and fasting glucose level were found between the single-bout and multiple-exercise subgroups in the OLETF Ex group, 15 and 1393 rats were required in each subgroup, respectively. Further research with a large sample size is warranted to establish suitable exercise prescriptions according to the pathophysiology of specific metabolic diseases.
